# The Relationship between the Expression of Ethylene-Related Genes and Papaya Fruit Ripening Disorder Caused by Chilling Injury

**DOI:** 10.1371/journal.pone.0116002

**Published:** 2014-12-26

**Authors:** Yuan Zou, Lin Zhang, Shen Rao, Xiaoyang Zhu, Lanlan Ye, Weixin Chen, Xueping Li

**Affiliations:** State Key Laboratory for Conservation and Utilization of Subtropical Agro-bioresources/Guangdong Provincial Key Laboratory for Postharvest Science and Technology of Fruits and Vegetables, College of Horticulture, South China Agricultural University, Guangzhou, 510642, P.R. China; Institute of Genetics and Developmental Biology, Chinese Academy of Sciences, China

## Abstract

Papaya (*Carica papaya* L.) is sensitive to low temperature and easy to be subjected to chilling injury, which causes fruit ripening disorder. This study aimed to investigate the relationship between the expression of genes related to ethylene and fruit ripening disorder caused by chilling injury. Papaya fruits were firstly stored at 7°C and 12°C for 25 and 30 days, respectively, then treated with exogenous ethylene and followed by ripening at 25°C for 5 days. Chilling injury symptoms such as pulp water soaking were observed in fruit stored at 7°C on 20 days, whereas the coloration and softening were completely blocked after 25 days, Large differences in the changes in the expression levels of twenty two genes involved in ethylene were seen during 7°C-storage with chilling injury. Those genes with altered expression could be divided into three groups: the group of genes that were up-regulated, including *ACS1/2/3*, *EIN2*, *EIN3s/EIL1*, *CTR1/2/3*, and *ERF1/3/4*; the group of genes that were down-regulated, including *ACO3*, *ETR1*, *CTR4*, *EBF2*, and *ERF2*; and the group of genes that were un-regulated, including *ACO1/2*, *ERS*, and *EBF1*. The results also showed that pulp firmness had a significantly positive correlation with the expression of *ACS2*, *ACO1*, *CTR1/4*, *EIN3a/b*, and *EBF1/2* in fruit without chilling injury. This positive correlation was changed to negative one in fruit after storage at 7°C for 25 days with chilling injury. The coloring index displayed significantly negative correlations with the expression levels of *ACS2*, *ACO1/2*, *CTR4*, *EIN3a/b*, *ERF3* in fruit without chilling injury, but these correlations were changed into the positive ones in fruit after storage at 7°C for 25 days with chilling injury. All together, these results indicate that these genes may play important roles in the abnormal softening and coloration with chilling injury in papaya.

## Introduction

Papaya (*Carica papaya* L.) fruit exhibits rapid softening and yellowing and has a short-term shelf life due to its climacteric behavior [1]. The storage of papaya fruit at low temperature is a useful practice for extending its commercial shelf life. However, papaya fruit is highly sensitive to chilling injury. Storing of them at an inappropriately low temperature results in skin scald, hard lumps in the pulp around the vascular bundles, water soaking of flesh, increased susceptibility to postharvest pathogens and abnormal ripening [2]. Ethylene not only regulates fruit ripening but is also involved in the cold stress response [3]. For example, low temperature stress induces ethylene production in eggplant, kiwifruit and mandarin [4]–[6]. Treatment of fruit with exogenous ethylene could alleviate chilling symptoms on the immature mangoes and nectarines [7], [8]. However, whether ethylene is related to abnormal softening and coloration of papaya fruit under cold stress requires further investigation.

The ethylene biosynthetic pathway is established as follows: methionine → S-adenosylmethionine (*SAM*) → 1-Aminocyclopropane-1-carboxylic acid (*ACC*) →ethylene [9]. In Arabidopsis (*Arobidopsis thaliana*), an important model system used for identifying genes and determining their functions, mutation of *ethylene overproducer1* or treatment with *ACC*, an ethylene precursor, could decrease the freezing-tolerance, whereas application with aminoethoxyvinyl glycine (AVG), an inhibitor of ethylene biosynthesis, or with Ag^+^, a perception antagonist caused the opposite effects [10]. Chilling injury to maize could increase *ACC* content [11]. Based on the separation and genomic research on the model plant such as *Arabidopsis*, tobacco related-mutants, a linear ethylene signal transduction model has been established: ethylene → receptors → *CTRs* → *EIN2* → *EIN3/EILs* → *ERF*. Ethylene molecules are perceived by a five-member receptor family, including *ETR1*, *ETR2*, *ERS1*, *ERS2* and *EIN4* [12]. The downstream signal transduction members include constitutive triple response (*CTRs*),ethylene insensitive 2 (*EIN2*), transcription factor families of *EIN3/EILs* and ethylene response factors (*ERFs*) [13]. In the absence of ethylene, *EIN3/EILs* is degraded through an ubiquitin-proteasome pathway mediated by EIN3-Binding F-box 1/2 (*EBF1/2*) [14]. Ethylene-insensitive mutants, including *etr1-1*, *ein4-1*, *ein2-5*, *ein3-1*, and *ein3eil1*, have a capability of freezing-tolerance. By contrary, ethylene-oversensitive mutants *ctr1-1* and *EIN3*-overexpressing plants have a lower capability of freezing-tolerance [10]. *ERF* has been shown to play an important role during fruit ripening and in response to stress such as low temperature [15]. Accumulation of *TERF2/LeERF2* mRNA in tomato was induced by cold, and over-expression of *TERF2/LeERF2* in tobacco enhanced the capability of freezing-tolerance [16]. Over-expression of the C-repeat binding factor (*CBF*), a member of the *ERF* transcription factor family, could increase the capability of freezing-tolerance [17]. On the contrary, knocking down *CBF1/3* with RNA interference (RNAi) and its-antisense lines in *Arabidopsis* decreased the capability of freezing-tolerance [18]. In the recent years, studies on ethylene related genes in response to low temperature in fruit have been reported. For example, *MdACO* and *MdACS* in apple (*Malus domestica Borkh*, *cv. Braeburn*) [19], *PpACO1*, *PpACS1*, *PpCTR1 and PpEIN2* in peach (*Prunus persicaL. Batsch*) [20], *EjETR1*, *EjCTR1 and EjEIL1* in loquat (*Eriobotrya japonica Lindl.*) [21], all showed the increased expression patterns in response to low temperature. In the contrast, the expression levels of *AdERS1b* and *AdETR1* genes in kiwifruit were reduced by low temperature [22]. Unfortunately, little information about the expression profiles of the genes related to ethylene biosynthesis and perception in response to low temperature stress in papaya fruit has been available.

The aim of the present study was to analyze expression profiles of the genes associated with ethylene biosynthesis and perception under low temperature stress and during ripening of papaya fruit treated with exogenous ethylene at room temperature (25°C). This study can expand our understanding of the functional roles of ethylene in the abnormal ripening of papaya fruit under low temperature stress.

## Materials and Methods

### Ethics statement

No specific permits were required for the described field studies. We confirm that the location is not protected in any way and also confirm that the field studies did not involve endangered or protected species.

We are sorry for some omits in our manuscript. Actually there are no animal involves in this study. All authors are linked to the same affiliation as described in this manuscript.

### Plant materials and treatments

Papaya fruit (*Carica papaya* L. cv. ‘Suiyou-2’) at color break stage (5% <peel colour <15% yellow) [23] were harvested from an orchard near Guangzhou, South China, then transported to the laboratory and selected. The selected fruits were first washed with water, dipped in 0.2% (w/v) hypochloride solution for 10 min to astringe cut and then soaked in 500 mg/L mixture solution of iprodione and prochloraz for 1 min to eliminate potential microbes. The fruits were divided into three groups for different treatments after natural drying. Two groups were placed into unsealed plastic bags and transferred to 7°C and 12°C for preservation, respectively. After storage for 25 and 30 days, fruits were removed from 7°C to room temperature (25°C), respectively, and treated with exogenous ethylene (ethrel) (1000 mg/L) for 1 min. The fruits stored at 12°C were treated the same as those fruits stored at 7°C. The third group of fruits was directly dipped into exogenous ethylene aqueous solution (1000 mg/L) for 1 min after harvest and storage at 25°C for ripening and used as the control group. Fruit samples stored at 12°C and 7°C were collected at 0, 10, 20, 25 and 30 days, respectively, and those treated with exogenous ethylene were collected at 1, 3 and 5 days after treatment. All the experiments were performed using 3 biological replicates. All the collected samples were quickly frozen in liquid N_2_ and stored at −80°C until further use.

### Fruit firmness, respiration, ethylene production and coloring index

Fruit firmness was measured using an Instron Harness Tester 5542 (Instron, Norwood, NJ, USA), fitted with an 8 mm diameter head. Penetration tests were run at a rate of 300 mm/min. After removal of 1 mm thickness of peel, firmness was measured on each of 4 fruits per treatment and at each time point, at 5 positions at the fruit equator [24].

Five papaya fruits per replicate were sealed in a 9 L-container and incubated for 2 h at storage temperature. Gas sample (1.0 ml) was withdrawn from the headspace for determination of the concentrations of C_2_H_4_ and CO_2_ using a Hitachi G3900 Gas Chromatograph (Hitachi America, Ltd., Brisbane, CA, USA) equipped with a Porapak-Q column and thermal conductivity/flame ionization detectors (TCD/FID). The temperatures of the injector, detector and column were set at 140, 150 and 80°C, respectively [24].

The stages of ripeness were determined by fruit peel colour as described by Miller and McDonald [25]. The scale ranges from 1 to 6: 1, entirely green −5%; 2, 5–25% yellow; 3, 25–50% yellow; 4, 50–75% yellow; 5, ≥75% yellow; and 6, orange blush/yellow. The fruit coloring index was defined and calculated as follows: coloring index  =  ∑ (coloring grade × number of fruit)/total number of fruit.

### Total RNA isolation and Real-time PCR analysis

Total RNA was isolated from the frozen papaya fruit following the protocol described in our previous report [26]. DNase (TaKaRa, Japan) was used to eliminate the potential DNA contamination in total RNA. Two (2) µg DNA-free RNA was used for first-strand cDNA synthesis by ReverTra Ace qPCR RT kit (TOYOBO, Japan). The final cDNA products were diluted to concentration of 80 ng/µl for subsequent real-time qPCR. The PCR reaction mixture (total volume of 20 µl) comprised of 10 µl of BIO-RAD iTaq Universal SYBR Green Supermix (Bio-Rad, USA), 0.25 µM of each primer, 2 µl of diluted cDNA, and 7.5 µl of DEPC-treated H_2_O. For all genes, cycling conditions included an initial denaturation at 95°C for 1 min, followed by 40 cycles of 95°C for 15 s, 55°C for 30 s, and 72°C for 35 s, and finally completed by a melting curve analysis program. In addition, reverse transcription negative control was included for checking the potential genomic DNA contamination. Each RT-qPCR analysis was performed in triplicate and the mean value was used for RT-qPCR analysis. The relative expression of all genes was calculated to the method of 2^−ΔΔCt^.

The gene-specific primers of *CTRs*, *ERFs* and reference gene (*EIF*) were adopted from the previous studies [26]–[28]. The other primers for real-time PCR analysis were designed with Primer Premier 6.0, according to the sequences of UTR region. The sequences of all primers for RT-qPCR analysis were described in S1 Table.

### Statistical analysis

Experiments were arranged using a completely randomized design. Each sample time point for each treatment was comprised of three independent biological replicates. Data were analyzed by analysis of variance (ANOVA). The Duncan's multiple range tests were used to compare the differences among mean values. Data were plotted on figures as mean ± standard deviation (SD). The difference between groups with (*p*<*0.05*) was regarded as statistically significant. Least significant difference (LSD) at the 5% level was analyzed by SPSS 21.0 software.

## Results

### Physiological characterizations during storage and ripening of papaya fruit

**Figure 1:**
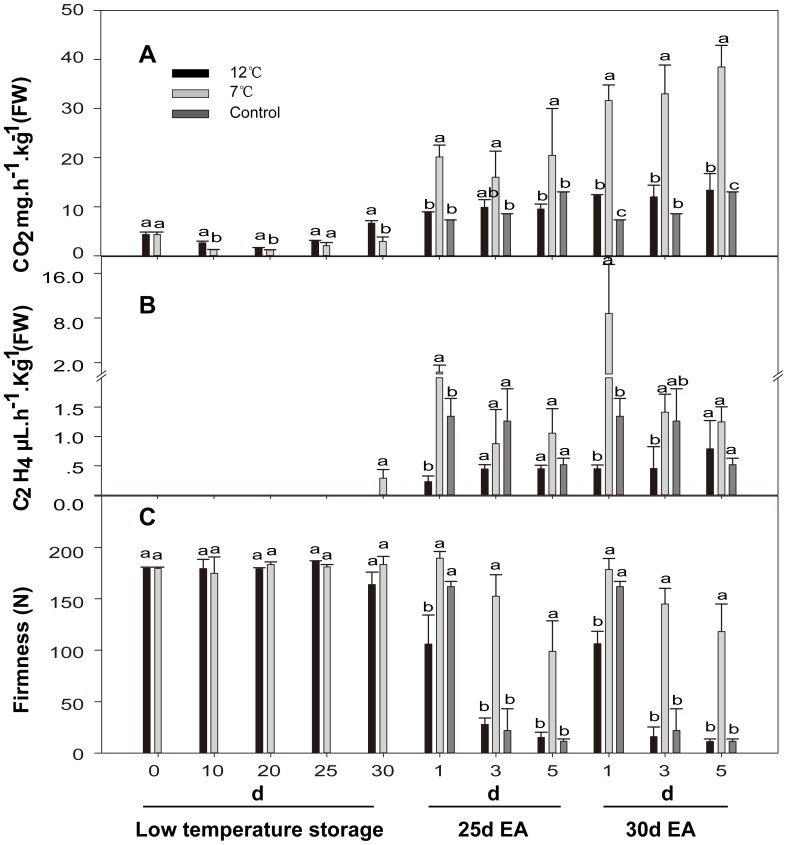
Respiration rate, ethylene production and pulp firmness of papaya fruit during low temperature storage and ripening at 25°C after exogenous ethylene treatment. A, respiration rate; B, ethylene production; and C, pulp firmness. 12°C, stored at 12°C; 7°C, stored at 7°C; 0 d 25°C EA, without low temperature-storage and directly treated with exogenous ethylene for ripening at 25°C; low temperature storage, at 12°C or 7°C storage; 25 d EA, treated with exogenous ethylene after 25 days at low temperature-storage; 30 d EA, treated with exogenous ethylene after 30 days at low temperature-storage. The values presented were obtained from three independent biological replicates. Mean values in graph followed by a different lower-case letters are significantly different at *P≦* *0.05* by Duncan's multiple range tests. Different letters were used to indicate the dates of all treatments on the same time, and different time points calculated separately. The LSD was done with all treatments.

**Figure 2:**
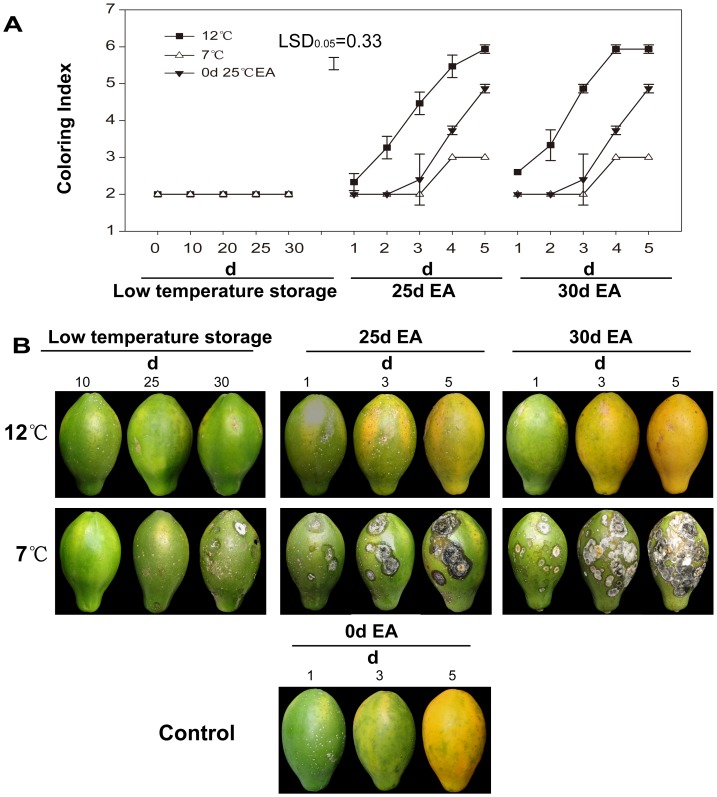
Coloring index and photographs of papaya fruit during low temperature storage and ripening at 25°C after exogenous ethylene treatment. A, coloring index; B, photographs. 12°C, stored at 12°C; 7°C, stored at 7°C; 0 d 25°C EA,without low temperature storage and directly treated with exogenous ethylene for ripening at 25°C; low temperature storage, at 12°C or 7°C storage; 25 d EA, treated with exogenous ethylene after 25 days at low temperature-storage; 30 d EA, treated with exogenous ethylene after 30 days at low temperature-storage. The values presented were obtained from 3 independent biological replicates.

In order to relate the expression of ethylene-related genes with papaya fruit ripening disorder caused by chilling injury, biological measurements including fruit respiration, pulp firmness, ethylene production and coloring index were conducted. These measurements revealed that the physiological changes were associated with chilling injury of papaya fruit (Figs. 1–2). The respiratory activity of papaya fruits decreased during 25 days of storage at 7 and 12°C, with the respiration rate lower than 7 mg·h^−1^·kg^−1^, but it showed a slight increase in 30^th^ day (Fig. 1A). An increase in respiratory activity of fruits in all treatment groups was observed on the first day after treatment with exogenous ethylene at 25°C, among which, the respiration rate of the fruits after storage at 7°C was increased most (Fig. 1A).

Papaya fruits did not produce any detectable ethylene during storage at 12 and 7°C, with values around 0.29 µL·h^−1^·Kg^−1^ until the 30^th^ day of storage at 7°C (Fig. 1B). The ethylene production of the control fruits and 7°C-storage fruits reached a peak on the first day after exogenous ethylene treatment at 25°C, and the ethylene production of fruits after storage at 7°C showed the highest level, while fruits after storage at 12°C showed the lowest one (Fig. 1B).

Papaya fruits did not lose firmness during storage at low temperature, and showed firmness values of 163.9 N and 183.3 N after 30 days of storage at 12°C and 7°C, respectively (Fig. 1C). Control fruits and 12°C-storage fruits showed a sharp decrease in pulp firmness and reached consumption firmness (<30 N) on the 3^rd^ day after exogenous ethylene treatment. However, 7°C-storage fruits still maintained a high firmness (around 120 N) on the 5^th^ day after exogenous ethylene treatment at 25°C (Fig. 1C), and the fruits finally cannot soften normally.

Papaya fruits showed little loss of green color during storage at low temperature. Fruits after 12°C storage achieved the same color as that of the control fruits on the fifth days after exogenous ethylene treatment at 25°C. However, storage at 7°C for 25 days completely blocked the coloration (Fig. 2A). Chilling injury symptoms such as skin scald, hard lumps and pulp water-soaking were observed in papaya fruit stored at 7°C on the 20^th^ day. The severity of chilling injury was increased by prolonging storage duration and by applying with exogenous ethylene at 25°C (Fig. 2B).

### Effect of low temperature storage on transcript levels of acyl-coenzyme A synthetases (*ACS*) and acyl-CoA oxidase (*ACO*)

**Figure 3:**
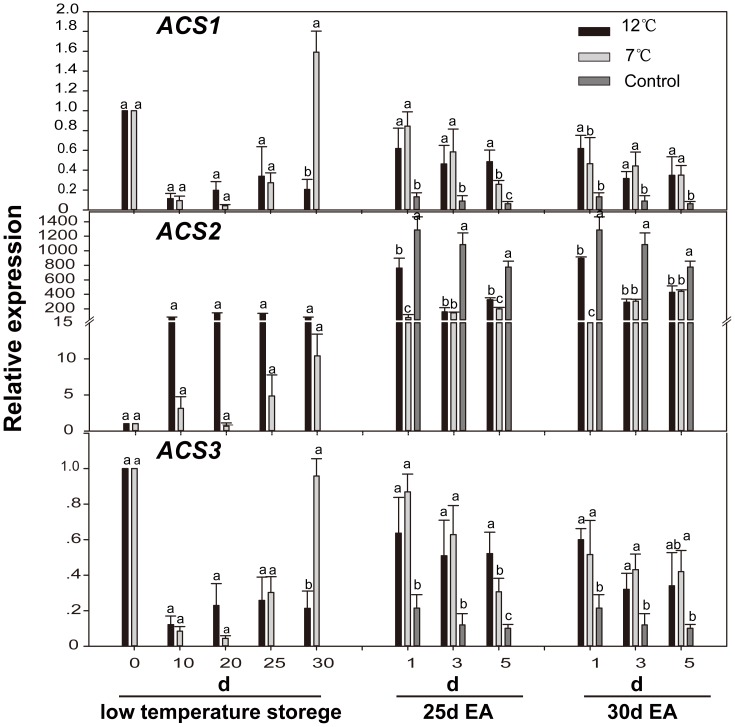
Expression levels of *CpACSs* in papaya fruit during low temperature-storage and ripening at 25°C after treatment with exogenous ethylene. Sampling details were the same as those labeled in Fig 1. The *y* axis represents the relative fold difference of mRNA level, which was calculated to the method of 2^−ΔΔCt^ using *CpEIF* as references gene. The values presented were obtained from 3 independent biological replicates. Samples from Day 0 (assigned an arbitrary quantity of “1”) were used as a calibrator to calculate the relative quantity of the results. Mean values in graph followed by a different lower-case letter are significantly different at *P*≦*0.05* by Duncan's multiple range tests. Different letters were used to indicate the dates of all treatments on the same time, and different time points calculated separately. The LSD was done with all treatments.

Effect of low temperature storage and exogenous ethylene on transcript levels of three *ACSs* and three *ACOs* were investigated. Under all conditions, *ACS1* and *ACS3* displayed a similar expression pattern and their expression levels were significantly decreased during storage at low temperature as compared with those on Day 0. Interestingly, fruits stored at 7°C showed a sharp increase on the 30^th^ day (Fig. 3). In contrast, a significantly increased accumulation of *ACS2* mRNA was found during storage at 12 and 7°C, and the former was obviously higher than the latter. The expression of *ACS1/3* in fruit after 7°C storage and control fruit was down-regulated on the 1^st^ day after exogenous ethylene treatment at 25°C, while their expression in fruit after 12°C storage was up-regulated. The accumulation of *ACS2* mRNA in all fruits was strongly induced by exogenous ethylene (Fig. 3).

**Figure 4:**
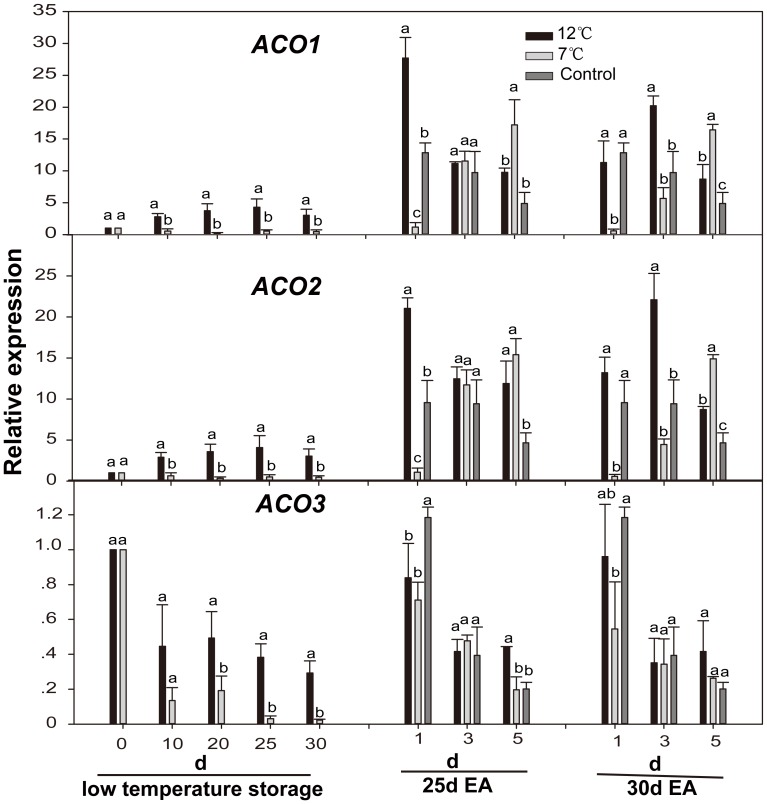
Expression levels of *CpACOs* in papaya fruit during low temperature-storage and ripening at 25°C after treatment with exogenous ethylene. Sampling details were the same as those labeled in Fig 1. The details for quantitative real-time PCR details are as described in Fig 2.

The expression patterns of *ACO1* and *ACO2* were similar during low temperature-storage period and ripening period. An increased accumulation of *ACO1/2* mRNA was seen during storage at 12°C. However, the expression of the *ACO1/2* was inhibited by storage at 7°C. The *ACO1/2* expression levels in the fruits with or without low temperature storage were obviously induced by exogenous ethylene, but reached a peak values in different times, i.e. at 12°C and 25°C on the 1^st^ day, at 7°C on the 3^rd^ day (Fig. 4). Under all conditions, the expression of *ACO3* gene was inhibited by low temperature-storage but induced by exogenous ethylene (Fig. 4).

### Effect of low temperature-storage on transcript levels of genes related to ethylene perception and signal transduction

**Figure 5:**
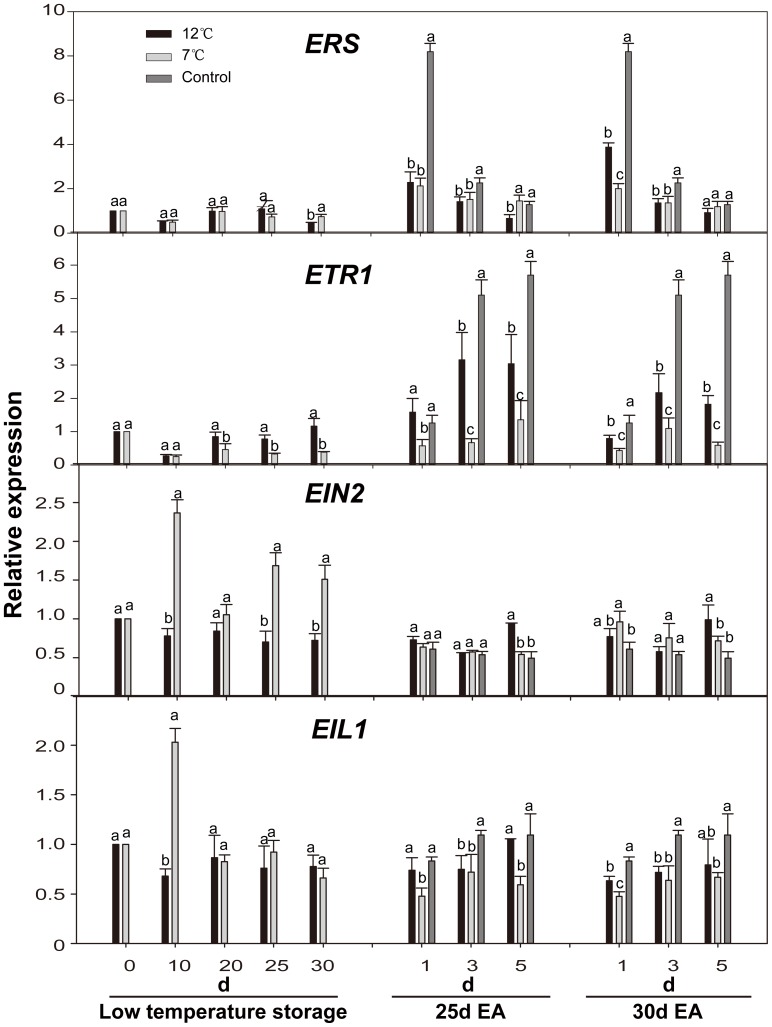
Expression levels of *CpERS*, *CpETR1*, *CpEIN2* and *CpEIL1* in papaya fruit during low temperature-storage and ripening at 25°C after treatment with exogenous ethylene. Sampling details were the same as those labeled Fig 1. The details for quantitative real-time PCR details are as described in Fig 2.

**Figure 6:**
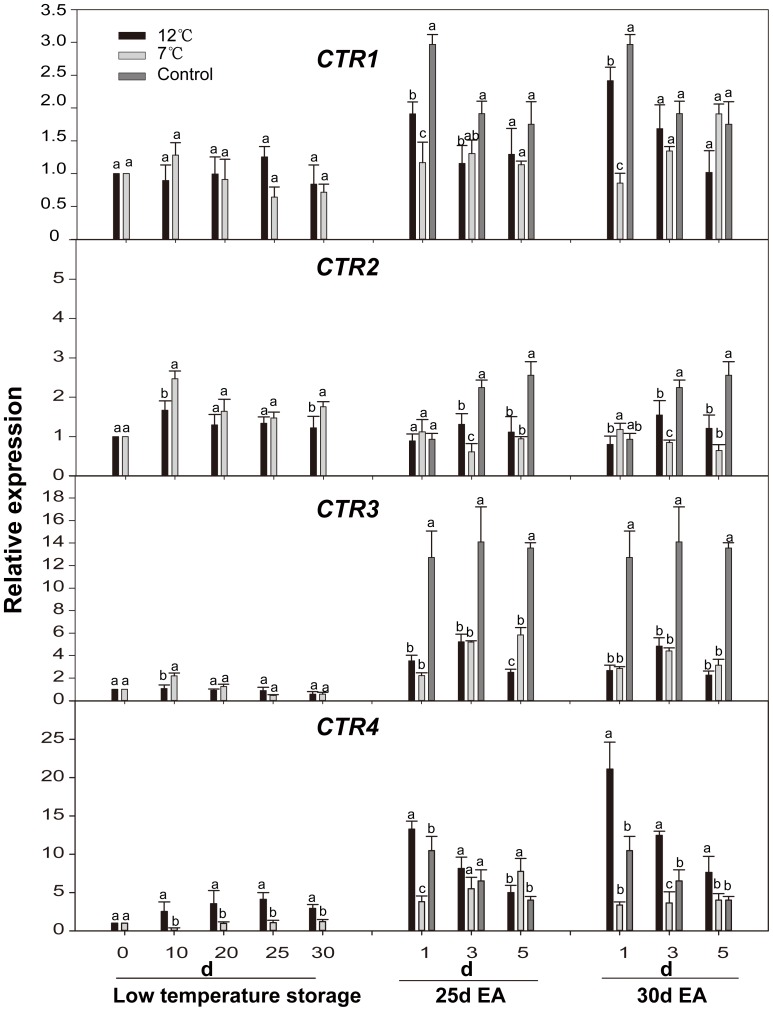
Expression levels of *CpCTRs* genes in papaya fruit during low temperature-storage and ripening at 25°C after ethylene treatment. Sampling details were the same as those labeled Fig 1. The details for quantitative real-time PCR details are the same as those described in Fig. 2.

**Figure 7:**
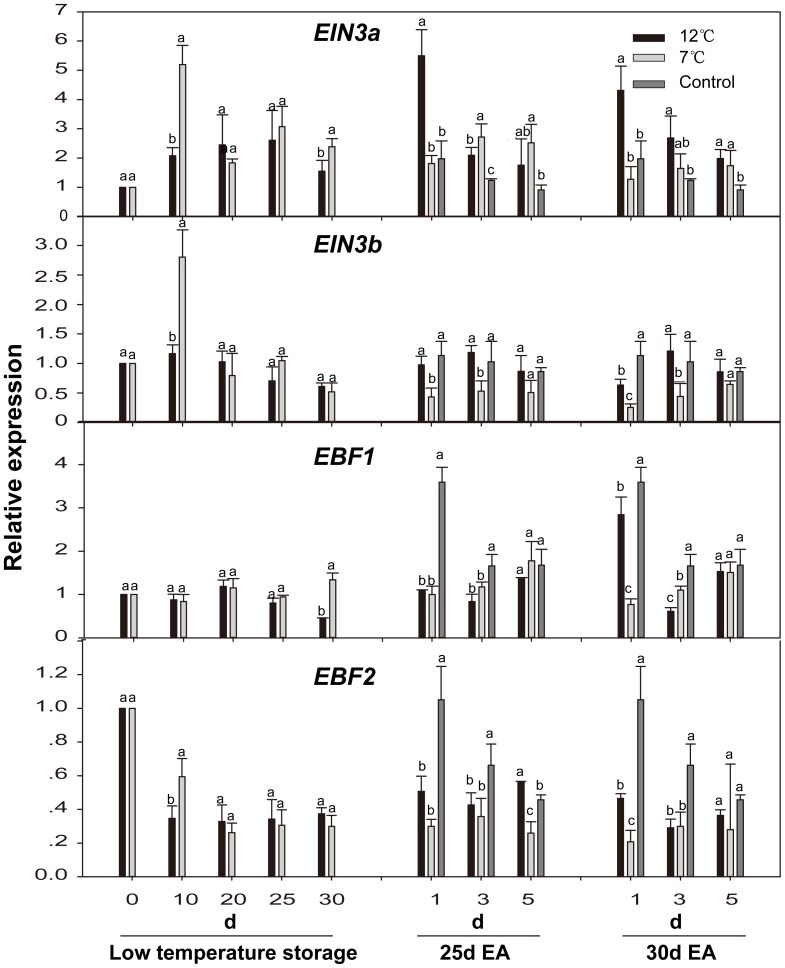
Expression levels of *CpEIN3s*(*CpEIN3a* and *CpEIN3b*)and the EIN3 binding F-box (*CpEBF1* and *CpEBF2*) genes in papaya fruit during low temperature-storage and ripening at 25°C after treatment with exogenous ethylene. Sampling details were the same as those labeled Fig 1. The details for quantitative real-time PCR were the same as those described in Fig 2.

**Figure 8:**
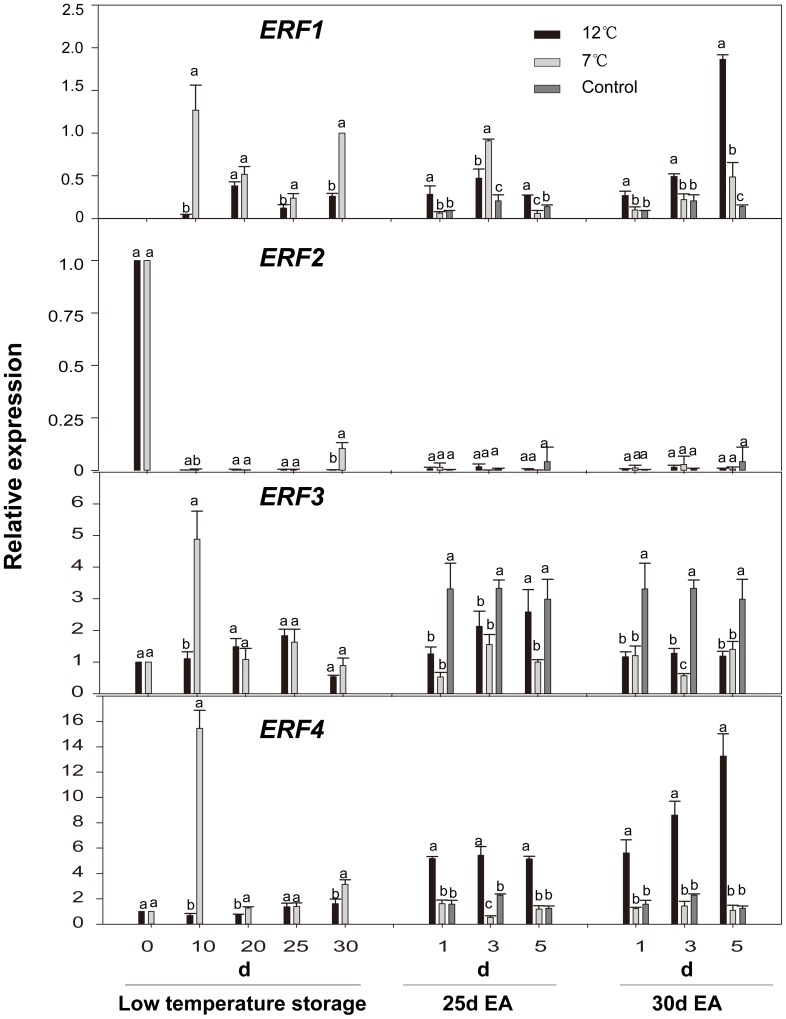
Expression levels of *CpERFs* (*ERF1*∼*4*) in papaya fruit during low temperature-storage and ripening at 25°C after treatment with exogenous ethylene. Sampling details were the same as those labeled Fig 1. Quantitative real-time PCR details are the same as those described in Fig 2.

Sixteen genes related to ethylene receptors and ethylene signal transductions in papaya fruit were selected and their expression patterns in responding to low temperature and exogenous ethylene treatment were analyzed. The results were shown in Figs. 5–8. The expression levels of ethylene receptor genes *ETR1* and *ERS* showed little change during storage at low temperature and there was no significant difference in *ERS* mRNA accumulation between the 12°C-storage fruit and the 7°C-storage fruit. The accumulation of *ETR1* mRNA in the 12°C-storage fruit was slightly higher than that in the 7°C-storage fruit in the late stage of storage (Fig. 5). Treatment with exogenous ethylene induced the expression of both *ERS* and *ETR1* genes in fruits with or without low temperature storage, among which, the accumulation of *ERS* mRNA in the control fruits reached a peak on the 1^st^ day, approximately 8 times that on Day 0, and the accumulation of *ETR1* mRNA in all the treated fruits reached a peak on the 5^th^ day during ripening (Fig. 5).

Expression levels of *CTRs* were increased to different degrees during low temperature storage period. Among which, the accumulation of *CTR2* showed the most obvious increase with a peak occurring on the 10^th^ day during storage at 12°C and 7°C, and the increase of 7°C-storage fruit was higher than that of 12°C-storage fruit. The abundance of *CTR1* in the fruit stored at 12°C and 7°C reached a peak on the 25^th^ and 10^th^ day, respectively. Expression level of *CTR3* reached a maximum on the 10^th^ day at 7°C-storage. However, the expression of *CTR4* was up-regulated only in the 12°C-storage fruit. Under all conditions, the expression of *CTR1/3/4* was strongly induced by exogenous ethylene. *CTR2* expression in control fruits was also induced by exogenous ethylene, and reached a peak on the 5^th^ day. However, its expression was repressed by exogenous ethylene after low temperature-storage (Fig. 6).

Expression level of EIN2 in fruit stored at 12°C was slightly lower than that on Day 0, and showed no significant changes during the storage at low temperature and fruit ripening at 25°C (Fig. 5). However, there was a peak of *EIN2* mRNA accumulation on the 10^th^ day during storage at 7°C, which showed 2.4 times higher than that on Day 0. The expression levels of *EIN2* gene in the control fruits and the 7°C storage-fruit were repressed by exogenous ethylene and remained constant during fruit ripening at 25°C (Fig. 5). Similar effects of low temperature and exogenous ethylene on *EIL1* mRNA accumulation were also found (Fig. 5).

The accumulation of *EIN3a* mRNA in fruit stored at 12°C and 7°C showed peaks on the 25^th^ and 10^th^ day, respectively, which was similar to *CTR1*. Expression of *EIN3b* reached a maximum on the 10^th^ day during storage at both 12°C and 7°C, and the expression of *EIN3b* at 12°C was remarkably lower than that at 7°C,which was similar to that of *CTR2*. The expression of *EIN3a* gene in the control fruits and 12°C-storage fruits was strongly induced by exogenous ethylene. However, its expression level in the fruits after 7°C storage was repressed. The accumulation of *EIN3b* mRNA in the control fruits and 12°C-storage fruits was slightly induced by treatment with exogenous ethylene, while that in 7°C-storage fruits showed no difference (Fig. 7).

Expression levels of *EBF* members showed little change during storage at low temperature, but the expression level of *EBF2* was significantly lower than that on Day 0. Expression of *EBF1* was strongly induced by exogenous ethylene in the control fruits and slightly induced in the fruits after 12°C storage. The expression of *EBF2* gene in the control fruits and the fruits after 12°C storage was slightly induced by exogenous ethylene. The accumulation of *EBF1* mRNA in the fruits after 7°C storage showed a gradual increase during fruit ripening. However, little change in the expression of *EBF2* gene in 7°C storage-fruit during fruit ripening was found (Fig. 7).

The expression of *ERF1/3/4* showed an obvious peak on the 10^th^ day of storage at 7°C. The accumulation of *ERF1/4* mRNA was promoted by exogenous ethylene after storage at 12°C, while in fruits after storage at 7°C, the accumulation of *ERF1/4* mRNA was just opposite. With exogenous ethylene treatment, the accumulation of *ERF3* mRNA in the control fruits was significantly higher than that of the low temperature storage-fruit. However, the expression level of *ERF2* was maintained at a very low but and constant level during storage at low temperature and ripening, which was significantly lower than that on the Day 0 (Fig. 8).

### Relationships between physiological characterizations and the expression of ethylene-related genes

In this study, we further investigated how the changes in expression levels of ethylene-related genes were related to the physiological characteristics including pulp firmness and coloring index during storage at low temperature and ripening. The results were presented in Tables 1–2. The relationship between pulp firmness and expression of ethylene related genes was quite different from the relationship between coloring index and expression of ethylene-related genes. Significantly positive correlations of pulp firmness of papaya fruit with expression levels of *ACS1*, *ACS2*, *ACS3*, *ACO1*, *ACO3*, *ERS*, *CTR1*, *CTR4*, *EIN2*, *EIN3a*, *EIN3b*, *EBF1*, and *EBF2* genes in the control fruits were observed. Among which, the correlations of pulp firmness of papaya fruit with expression levels of *ACS2*, *ACO1*, *ACO2*, *CTR1*, *CTR4*, *EIN3a*, *EIN3b*, and *EBF1* in the 7°C-storage fruits with chilling injury were changed into negative ones, and the expression levels of *ACS1*, *ACS3*, *ACO3*, *ERS*, and *EIN2* in fruit stored at 7°C were reduced. On the other hand, pulp firmness of papaya fruit had highly negative correlations with the expression levels of *ETR1*, *CTR2*, *CTR3*, and *EIL1* in the control fruits. Among which, in the fruits stored at 7°C with chilling injury, the correlation between pulp firmness of papaya fruit and the expression level of *CTR2* gene was changed into the positive correlation whereas the coefficient for the negative correlations between pulp firmness of papaya fruit and the expression levels of other genes showed a slight decrease.

**Table 1:** Correlation between papaya fruit pulp firmness and expression levels of ethylene-related genes.

Correlation coefficients/R											
	*ACS1*	*ACS2*	*ACS3*	*ACO1*	*ACO2*	*ACO3*	*ETR1*	*ERS*	*CTR1*	*CTR2*	*CTR3*
**25°C**	.349**	.867*	.995*	.834*	.573	.992**	−.998**	.998**	.998**	−.993**	−.891*
**12°C**	.888*	.902*	.806	.481	.325	.977**	−.723	.866*	.847*	−.826*	−.153
**7°C**	.846*	−.679	.835*	−.931**	−.897*	.966**	−.696	−844*	−.486	.542	−.686
	*CTR4*	*EIN2*	*EIL1*	*EIN3a*	*EIN3b*	*EBF1*	*EBF2*	*ERF1*	*ERF2*	*ERF3*	*ERF4*
**25°C**	.946**	.947**	−.998**	.972**	.833*	.997**	.960**	−.780	−.605	.527	−.142
**12°C**	.798	−.130	−.537	.942**	−.471	.543	.402	−.468	−.296	−.452	−.501
**7°C**	−.690	.452	−.587	−.457	−.660	−.933**	−.011	−.117	.399	−.291	.284

*, significant at *P*≦*0.05*;

**, significant at *P*≦*0.01*.

Correlations were based on n = 18 (three replicates × six sampling time points (after exogenous ethylene treatment)).

12°C, stored at 12°C; 7°C, stored at 7°C; 25°C, without low temperature storage and directly treated with exogenous ethylene for ripening at 25°C.

**Table 2:** Correlation between coloring index of papaya fruit coloring index and expression levels of ethylene-related genes.

Correlation coefficients/R											
	*ACS1*	*ACS2*	*ACS3*	*ACO1*	*ACO2*	*ACO3*	*ETR1*	*ERS*	*CTR1*	*CTR2*	*CTR3*
25°C	−.860*	−.962**	−.728	−.964**	−.944**	−.744	.702	−.706	−.701	.742	.243
12°C	−.796	−.772	−.734	−.592	−.484	−.884*	.637	−.870*	−.832*	.644	−.130
7°C	−.705	.611	−.645	.846*	.811	−.778	.404	−.601	.522	−.320	.294
	*CTR4*	*EIN2*	*EIL1*	*EIN3a*	*EIN3b*	*EBF1*	*EBF2*	*ERF1*	*ERF2*	*ERF3*	*ERF4*
25°C	−.866**	−.864*	.611	−.815*	−.964**	−.601	−.842*	.040	.998**	−.987**	−.653
12°C	−.812*	.401	.668	−.938**	.222	−.347	−.236	.535	.015	.474	.537
7°C	.556	−.348	.273	.248	.643	.898*	−.222	−.078	−.508	.289	−.095

*, significant at *P*≦*0.05*;

**, significant at *P*≦*0.01*.

Correlations were based on n = 18 (three replicates × six sampling time points (after exogenous ethylene treatment)).

12°C, stored at 12°C; 7°C, stored at 7°C; 25°C, without low temperature storage and directly treated with exogenous ethylene for ripening at 25°C.

At the same time, the relationships between fruit coloring index and the expression levels of ethylene-related genes were also analyzed. The results showed that coloring index had a significantly positive correlation with the expression level of *ERF2* in the control fruits whereas the positive correlation was changed to the negative one correlation in the fruits stored at 7°C. On the contrary, synchronous changes revealed significantly negative correlations between coloring index of the control fruits and the expression levels of *ACS1*, *ACS2*, *ACO1*, *ACO2*, *CTR4*, *EIN2*, *EIN3a*, *EIN3b*, *EBF2*, and *ERF3* genes showed significantly negative correlations. The correlations between coloring index of the control fruits and the genes mentioned above were changed to the positive ones expect for *ACS1*, *EIN2* and *EBF2* whose levels were declined in the 7°C-storage fruit. Altogether, these results suggest that *ACS2*, *ACO1*, *ACO2*, *CTR1*, *CTR4*, *EIN3a*, *EIN3b*, and *EBF1* may play an important role in the abnormal softening with chilling injury in papaya fruit, and that *ACS2*, *ACO1*, *ACO2*, *CTR4*, *EIN3a*, *EIN3b*, and *ERF3* have close relationship with the poor color development of papaya fruit at the 7°C-storage.

## Discussion

Low temperature storage is a major post-harvest technology used commonly to extend the post-harvest life of fresh fruit and to delay fruit ripening [29]. However, low temperature-storage of papaya, the chilling-sensitive fruit, frequently induces chilling injury [2], and may cause unfavorable effects on ethylene biosynthesis, perception and signal transduction mechanisms. Previous studies mainly focused on the changes of ethylene biosynthesis during papaya fruit ripening, few studies have been conducted on the expression of genes related to ethylene signaling transduction in papaya fruit ripening at room temperature (25°C) after being subjected to chilling injury and analyzed the relationship between the ethylene-related genes expression and papaya fruit ripening disorder caused by chilling injury. The present study investigated the changes and regulation of ethylene-related genes in papaya fruit at the mRNA level with focus on low temperature stress and ripening to gain a better understanding of the role of ethylene in papaya softening and coloration after chilling injury.

The results of this study showed that the coloration and softening of papaya fruits stored at 7°C for 25 days and even the fruit treated with exogenous ethylene at 25°C were completely blocked. When papaya fruit were transferred from 7°C to 25°C, much skin scald and water-soaking occurred on their peel. Small sunken dead spots developed, which served as the places of entrance for fungi. Within 2 to 5 days, the fruit was overrun with fungi, breakdown and proceeded without ripening, but the fruits stored at 12°C maintained good quality after low temperature-storage for 30 days.

*ACS* and *ACO* two key enzymes involved in the ethylene biosynthesis, have been isolated and extensively studied in papaya fruit. In the present study, three isoforms of *ACSs* (*ACS1*, *ACS2*, and *ACS3*) and three isoforms of *ACOs* (*ACO1*, *ACO2*, and *ACO3*) were detected (Figs. 3–4). Interestingly, a significant decrease in the expression of *ACS1* and *ACS3* was observed during low temperature storage and there was a sharp increase on the 30^th^ day during storage at 7°C. However, the expression patterns of *ACS2* just showed the opposite, i.e. its expression was induced by storage at 12°C and 7°C. In addiction, *ACS2* gene expression in the 7°C-storage fruits was much lower than in the fruit with other treatments during ripening at 25°C (Fig. 3), suggesting that *ACS2* is more sensitive to low temperature, with a coordinated response to the progress of papaya fruit ripening, which is markedly slowed down by low temperature stress during ripening. It has been shown that *ACS1* mRNA accumulation of banana is suppressed during 10°C storage [30]. In contrast, chilling-induced the expression of *ACS* has been found in apple [19] and citrus [31].

*ACS* and *ACO* genes in papaya were expressed differentially throughout the fruit ripening process. Base on their expression patterns, *ACSs* and *ACOs* are closely correlated to ethylene production and ethylene climacteric burst after exogenous ethylene treatment (Figs. 1, 3, and 4). This is supported by a recent report of the transgenic line of antisense-*ACOs*, which showed that the suppression of *ACOs* resulted in low levels of ethylene [32]. The expression of *ACO3* gene was suppressed during both 12°C- and 7°C- storage. The expression of *ACO1*/*2* genes was induced at appropriate temperature (12°C), but suppressed at 7°C, and its expression level in fruits subjected to chilling injury (7°C) was much lower than that in fruits with other treatments on the 1^st^ day after being treated with exogenous ethylene (Fig. 4). In peach, expression of *ACO1/2* genes was up-regulated during 12°C-storage [20]. However, the expression of *ACO1* gene in banana was decreased during low temperature-storage [33]. Their expression patterns suggest that *ACO1/2* genes are more closely associated with abnormal ripening of papaya fruit under low temperature stress, which is consistent with our results that 12°C-storage can delay papaya fruit ripening whereas 7°C-storage may completely block papaya fruit ripening.

Our results also showed that the accumulation of the *ETR1* and *ERS* transcripts remained low and constant during storage at low temperature (Fig. 5). An opposite expression pattern has been reported for *ETR1* genes in other plants, including pear [34] and tomato [35], whose mRNA levels were induced by low temperature stress. After being treated with exogenous ethylene, the expression levels of *ETR1* and *ERS* genes in the 7°C-storage fruits were much lower than in the fruits with other treatments (Fig. 5), suggesting that both *ETR1* and *ERS* participate in papaya fruit ripening. It was reported that the accumulation of *ETR1* mRNA in avocado [36], and apple [37], [38] was strongly induced by exogenous ethylene. Interestingly, little change in the accumulation of *ETR1* mRNA was seen in persimmon [39], and citrus [40] after exogenous ethylene treatment. Accumulation of *ERS* mRNA in avocado was up-regulated during low temperature storage [36].

In the ethylene signaling system, *CTRs* play a central role in regulating a negative ethylene response in the absence of ethylene [41]. The expression patterns of *CTRs* showed an increase to different degrees during low temperature-storage (Fig. 6). Similar expression pattern for an CTR-type gene has been observed in avocado [36], and kiwifruit [22]. After being treated with exogenous ethylene, only a slight difference in the expression of *CTR2/3* genes was seen between the 7°C-storage fruit and 12°C-storage fruit whereas the expression of *CTR1/3* genes in the 7°C-storage fruit was much lower than that in the 12°C-storage fruit (Fig. 6), suggesting that *CTR2/3* genes are relatively sensitive to chilling injury, but *CTR1/4* also have a closer relationship with papaya fruit ripening. This is supported by a recent report, which showed that *LeCTR2* was involved in defense and stress responses [42]. The genes of *CTRs* have been shown to be induced by exogenous ethylene in apple [38], grape [43], and banana [44]. It would be interesting to find out a negative regulator of ethylene signal system that is induced by ethylene during fruit ripening as it is supposed that in most tissues that produce large amounts of ethylene, the induction of the negative expression regulator may function as a damping mechanism to response and temper the suddenly increased ethylene concentration, in order to delay the fruit ripening and senescence process [35].

We also observed that accumulation of *EIN2* mRNA reached a peak on the 10^th^ day during 7°C-storage (Fig. 5). It is assumed that chilling injury may induce the accumulation of *EIN2* mRNA. A similar phenomenon was found in peach [19]. *EIN2* has been shown to act as a bi-functional transducer of ethylene and stress responses through mediating the signal propagation between *CTR* and *EIN3/EILs*, the downstream components [45]. The expression of *EIN2* gene in the all treated fruits showed a slight difference during fruit ripening (Fig. 5). The expression of *EIN2* was reduced by treatment with exogenous ethylene in apple [46], but was not affected by exogenous ethylene in tomato [47]. This disparity indicate that a feedback inhibition may be involved in the regulation of *CpEIN2* expression and the decrease in *CpEIN2* mRNA levels during fruit ripening after exogenous ethylene treatment is a consequence of the ethylene-induced senescence process in papaya fruit.

Our results showed that the expression of *EIL1* gene reached a peak on the 10^th^ day during 7°C-storage (Fig. 5). This result is similar to that observed in kiwifruit [22]. In rice [48], accumulation of *OsEIL1* and *OsEIL12* transcripts reached a maximum at 1 and 0.5 h after wounding, respectively, suggesting that *EIL* may be involved in stresses-response. *CpEIL1* gene showed no response to exogenous ethylene (Fig. 5), which is similar to that in Kiwifruit [22], [49] and muskmelon [50]. Unexpectedly, the expression of *EIL1* in ‘Taishanzaoxia’ apple was induced by exogenous ethylene [51]. In melon, *CmEIL1* and *CmEIL2* proteins were found to interact with and activate a *Cm-ACO1* promoter in the yeast one-hybrid system, thereby promoting *Cm-ACO1* expression and ethylene production. This cascade may result in a climacteric ethylene emission, which, in turn, further stabilizes *EIL1* protein [50].

The accumulation of both *EIN3a* and *EIN3b* mRNA showed a peak at 7°C storage (Fig. 7), suggesting that *EIN3a/b* may be involved in chilling stress response. A recent study with the transgenic line of *ein3-1* showed that this transgenic line faced higher oxidative stress under salinity than the wild-type did [52]. Being treated with exogenous ethylene, only the accumulation of *EIN3a/b* mRNA of papaya fruit stored at 7°C was decreased, whereas the accumulation of *EIN3a/b* mRNA in fruit of other-treatment groups was increased (Fig. 7). The accumulation of *EIN3* was not affected by treatment with exogenous ethylene in petunia [53] and miniature rose [54]. However, in peony, *EIN3* expression was decreased by treatment with exogenous ethylene [55]. Over-expression of *EIN3* in transgenic *Arabidopsis* plants resulted in a constitutive ethylene response [56]. It has been proposed that *EIN3* is involved in ripening and in responding to stress signals.

*EBF1* and *EBF2* have been shown to act on ethylene signal system by regulating *EIN3/EIL* turnover. Ethylene appears to block protein ubiquitination, resulting in the increase in *EIN3/EILs* protein level, which, in turn, mediates ethylene signaling [57]. Expression level of *EBF2* was significantly lower than that on Day 0, and showed no changes during low temperature storage (Fig. 7). In addition, exogenous ethylene induced the expression of *EBF1*/*2* genes, but 7°C-storage fruit showed the lowest (Fig. 7). A similar expression pattern was seen in tomato [58]. A recent study reported that the ethylene signal transduction pathway in *Arabidopsis* was controlled by a negative feedback regulation between *EBF2* and *EIN3*, where *EIN3* targets the promoter of *EBF2* to modulate its expression level and thus allows fine-tuning of ethylene response [57], [59]. In this model, ethylene elevates the level of *EIN3* protein, and the resulting accumulation of EIN3 induces the expression of *EBF2*, which promotes the degradation of *EIN3* and hence down-regulates ethylene signaling, allowing a rapid recovery after ethylene removal [59].

Accumulating lines of evidences have suggested that *ERF* proteins regulate the responses to a variety of biotic and abiotic stresses as well as the development of plant [60], [61]. This study showed that the expression of *ERF1/3/4* genes reached a peak on the 10^th^ day during 7°C-storage (Fig. 8). In most studies, *ERFs* have been shown to be induced at low temperature in plants including wheat [62] and tomato [16]. A recent report indicated that *ERF1* interacted with multiple *cis*-acting elements and activated the expression of the stress responsive-genes and the genes related to ABA biosynthesis, consequently causing the rise in ABA biosynthesis, and ultimately enhancing the tolerance toward high salinity and low temperature [16], [63]. Exogenous ethylene treatment induced the expression of *ERF1/3/4* genes but suppressed the expression of *ERF2* (Fig. 8). It has been shown that *ERFs* are induced by treatment with exogenous ethylene in plants, such as *LeERF1/4* in tomato [64]. *PsERF1a/b*, *PaERF3a/b*, and *PaERF12* in pear [65], and *ERF1/2* in apple [38]. However, *LeERF2/3* in tomato [64] were not affected by the treatment with exogenous ethylene.

In conclusion, this study has revealed that twenty two ethylene-related genes displayed different expression patterns in responding to low temperature stress and during ripening. While these ethylene-related genes are generally considered to be involved in low temperature stress and ripening, it is not yet clear to whether ethylene signaling plays an important role in ripening disorder of papaya fruit. This study investigated the expression profiles of genes related to ethylene signaling during papaya fruit ripening after cold stress to elucidate the role of these signal transduction components. Our subsequent study is to identify the novel and important proteins that interact with ethylene signal transduction components via the yeast two-hybrid screening using 2–4 key genes as bait to elucidate the signaling mechanism and to provide new strategies to control ripening disorder of papaya fruit.

## Supporting Information

S1 Fig **Dissociation curves data for the 22 genes related to ethylene synthesis, signaling and one reference gene.** All the dissociation curves for 22 genes related to ethylene synthesis, signaling and a reference gene showed single peaks. No amplicon was observed in No Template Control (NTC) as indicated by the red arrow.
(TIF)

S1 Table **Real-time PCR primers targeting genes related to ethylene synthesis and signaling transduction.**
(DOC)
