# Supplementary figures and images for: The Relationship between the Expression of Ethylene-Related Genes and Papaya Fruit Ripening Disorder Caused by Chilling Injury
Source: PLoS One. 2014 Dec 26;9(12):e116002. doi: 10.1371/journal.pone.0116002 (PMC4277447; doi:10.1371/journal.pone.0116002)

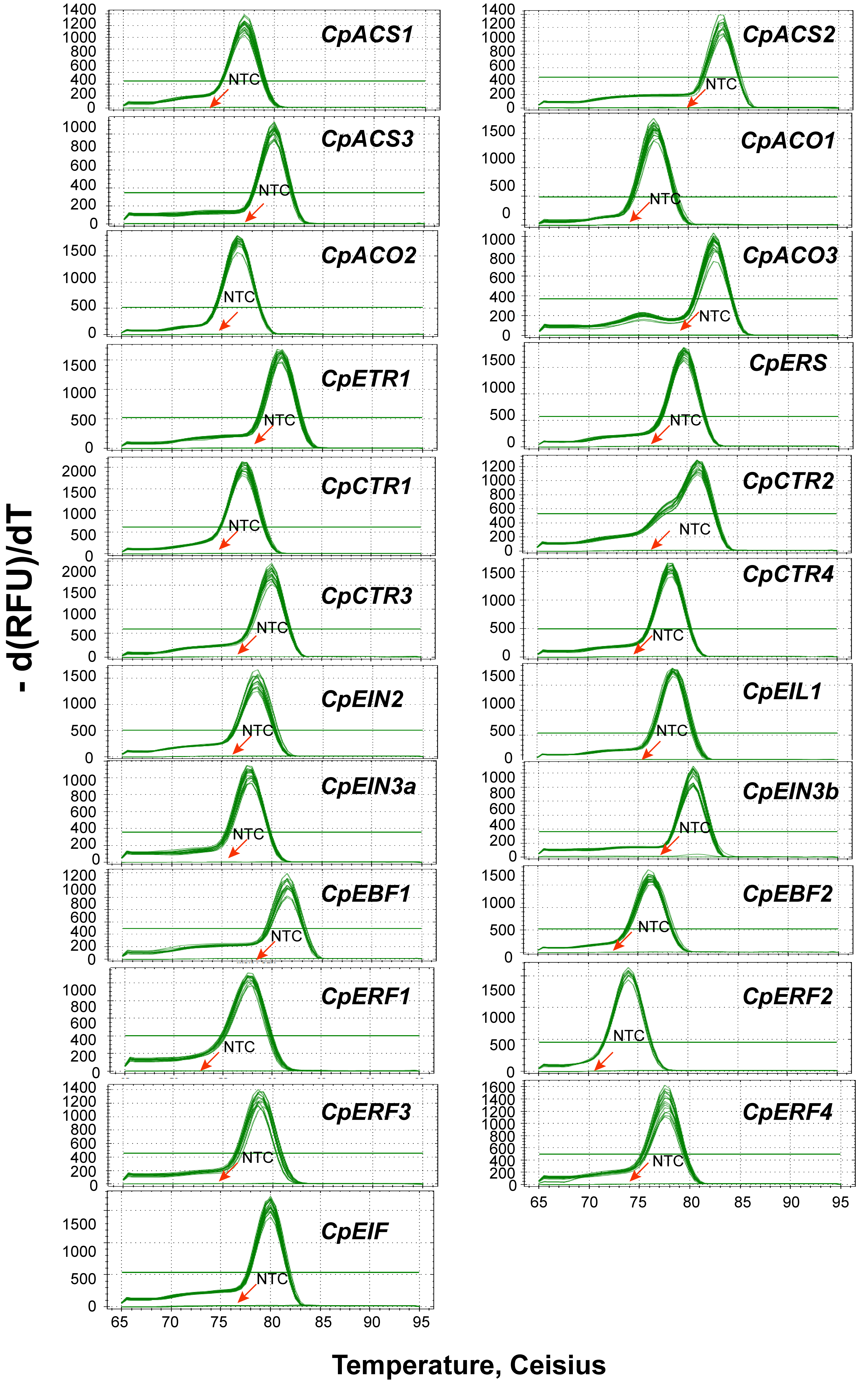

Supplement: S1 Fig — Dissociation curves data for the 22 genes related to ethylene synthesis, signaling and one reference gene. All the dissociation curves for 22 genes related to ethylene synthesis, signaling and a reference gene showed single peaks. No amplicon was observed in No Template Control (NTC) as indicated by the red arrow. (TIF) [file pone.0116002.s001.tif]
