# Supplementary material for: The Relationship between the Expression of Ethylene-Related Genes and Papaya Fruit Ripening Disorder Caused by Chilling Injury
Source: PLoS One. 2014 Dec 26;9(12):e116002. doi: 10.1371/journal.pone.0116002 (PMC4277447; doi:10.1371/journal.pone.0116002)
Supplement: S1 Table — Real-time PCR primers targeting genes related to ethylene synthesis and signaling transduction. (DOC) [file pone.0116002.s002.doc]

**Table 1S.** Real-time PCR primers targeting genes related to ethylene synthesis and signaling transduction.

| Gene | Accession | Forward primer(5'-3') / Reverse primer(5'-3') |
| --- | --- | --- |
| ACO1 | AJ605321.1 | AGCCAATCAACTTCCAAACACC/ AGTCTACTGTAACTCCTGGTGCC |
| ACO2 | AF379855.1 | AAGCCAATCAACTTCCAAACAC/ AGTCTACTGTAACTCCTGGTGCC |
| ACO3 | L76283.1 | AATCTTCACCATAAAATACCCAAT/ GTTCGAGGATTACATGAAGCTG |
| ACS1 | U68216.1 | CGAATCATCACCCAACGAATC/ TGTCTCCTCACTCGCCTATACC |
| ACS2 | AM113989.1 | CACCACCTTAGACTCACA/GACGAGACCAACCCAAAG |
| ACS3 | Y11357.1 | AACTAGATCGCCTGATGCACG/ATGTCTCCTCACTCGCCTATACC |
| ETR1 | KF709952 | GAGGTTGTTGCGGTTCGTGT/ AGAGCAACCGCCACCTGAT |
| ERS | AF311942.1 | GCAGGTTAAGGATTCTGGATGTGGTA/CTTTGTCCGCACCCTCACTTTCT |
| CTR1 | JN546578 | GCAGGTTAAGGATTCTGGATGTGGTA/CTTTGTCCGCACCCTCACTTTCT |
| CTR2 | KC991147 | GATCCCAACTTACGCCCA/CTTCCATCCAGCCCTTTT |
| CTR3 | KC991148 | TCGGATGTCGCAGTGAAGG/CTGTCCAATTCTTGTCCACCAAT |
| CTR4 | KC991149 | CGGATGCCTAATGCTGGAGA/TCGCCGACCTTAACTGTATATGTA |
| EIN2 | KF709946 | CTCATCAACACAGGTCAAG/TAGCAGCATAGCAGAAGTT |
| EIL1 | KF709947 | CAGCATCACCAGGACCAA/AAGCCAAATCAAAGGGAGAC |
| EIN3a | KF709948 | GGCTCAACAGTCAGATGG/CTCCAACAATCTCATTCTCA |
| EIN3b | KF709949 | CATCACCAGGACCAATACTTC/GTGTCCATCCTACTGCTG |
| EBF1 | KF709950 | TGCTTAGTCCCTTTCCATC/GCTCACACTCTCCAGAAG |
| EBF2 | KF709951 | GACACAGCCATTCTATTACCTT/CTCAGGGAAACACGACCAT |
| ERF1 | JX532988 | TCTCCGCCGTCTGATGATT/GCAATATCCAGTTTCACACCAT |
| ERF2 | JX532989 | ATGTCTCTCTTCGCTTCA/CCCATCTCTTCACTATCC |
| ERF3 | JX532990 | GAAGAGGAGGAGGATGGTGAT/CAGTTAAGCCACTCTTATCTATCC |
| ERF4 | JX532991 | GAGAGCAAGATAATACAGGC/GTTAGGGAGAAAAACAAAAA |
| EIF | FJ644949.1 | AGGCAGGCAAGAGAAGAT/TTCATACCGAGTAGCGATTC |

**Fig 1S. Dissociation curves data for the 22 genes related to ethylene synthesis, signaling and one reference gene.** All the dissociation curves for 22 genes related to ethylene synthesis, signaling and a reference gene showed single peaks. No amplicon was observed in No Template Control (NTC) as indicated by the red arrow.
